# Zearalenone and Its Emerging Metabolites Promptly Affect the Rumen Microbiota in Holstein Cows Fed a Forage-Rich Diet

**DOI:** 10.3390/toxins15030185

**Published:** 2023-02-28

**Authors:** Thomas Hartinger, Iris Kröger, Viktoria Neubauer, Johannes Faas, Barbara Doupovec, Dian Schatzmayr, Qendrim Zebeli

**Affiliations:** 1 Institute of Animal Nutrition and Functional Plant Compounds, University of Veterinary Medicine, 1210 Vienna, Austria; 2 Biomin Research Center, Biomin Holding GmbH, 3430 Tulln, Austria

**Keywords:** dairy, *Fusarium*, mycotoxin, rumen, sequencing

## Abstract

The study investigated the short-term effects of a single oral bolus of zearalenone (ZEN) on the rumen microbiota and fermentation patterns in four rumen-cannulated Holstein cows fed a forage diet with daily 2 kg/cow concentrate. During the baseline day, cows received uncontaminated concentrate, followed by ZEN-contaminated concentrate on the second day, and again the uncontaminated concentrate on day three. Free rumen liquid (FRL) and particle-associated rumen liquid (PARL) were collected at different hours post-feeding on all days to analyze the prokaryotic community composition, absolute abundances of bacteria, archaea, protozoa, and anaerobic fungi, as well as short-chain fatty acid (SCFA) profiles. The ZEN reduced the microbial diversity in FRL but not in the PARL fraction. The abundance of protozoa was higher after ZEN exposure in PARL, which may be related to their strong biodegradation capacity that, therefore, promoted protozoal growth. In contrast, α-zearalenol might compromise anaerobic fungi as indicated by reduced abundances in FRL and fairly negative correlations in both fractions. Total SCFA significantly increased in both fractions after ZEN exposure, while the SCFA profile only changed marginally. Concluding, a single ZEN challenge caused changes in the rumen ecosystem soon after intake, including ruminal eukaryotes, that should be the subject of future studies.

## 1. Introduction

Mycotoxins constitute serious contaminants in ruminant feeds leading to impaired animal health and welfare as well as economical losses due to reduced performance [1]. Among the vast variety of mycotoxins that are frequently observed in feedstuffs, *Fusarium* mold-derived zearalenone (ZEN) represents one of the main contaminants worldwide with severe consequences for ruminant livestock production [2]. Due to the estrogenic character of ZEN and its metabolites, the chronic exposure to this mycotoxin causes hyperestrogenism with false estrus, ovarian cysts, and reproduction failure being among the prominent symptoms in livestock animals [3]. Besides these well-studied chronic impairments, recent investigations provided evidence for the detrimental short-term effects of ZEN on both the rumen ecosystem and animal health. Indeed, a disturbed rumen ecosystem following the ZEN exposure was clearly indicated by decreased concentrations of total short-chain fatty acids (SCFA) as well as an altered bacterial community. Likewise, the onset of mild fever and a reduced eating time of cows suggested compromised animal health in response to 5 mg/d ZEN exposure [4].

However, the impact of mycotoxins on ruminants may also vary with different feeding regimes as the inclusion of high concentrate amounts reduces the mycotoxin degradation capacity of the rumen microbiota [2,5]—presumably because high-concentrate feeding itself represents a stressor, triggering acidotic and dysbiotic conditions in the rumen (e.g., Hua et al. [6]). Since the aforementioned findings on short-term implications of ZEN were obtained in cows fed substantial concentrate levels, i.e., 40% on a dry matter (DM) basis [4], it may be assumed that cows receiving forage-dominated diets possess a stronger resistance against mycotoxins due to the higher detoxification capacity of the rumen microbiota. Yet, in the case of ZEN, its rumen biodegradation often results in the more toxic ZEN-metabolites, such as α-zearalenol (α-ZEL; [7]). Therefore, it is of interest to evaluate the short-term effects of ZEN exposure in forage-fed cows, especially as forage-based milk production systems are worldwide common and also expanding in Europe [8], whereby mycotoxin contamination of forages and pastures is increasingly becoming an overall concern due to altered climatic conditions [9,10].

The present study aimed to investigate the short-term effects of a single ZEN dose on the microbiota and fermentation pattern in the rumen of dry Holstein cows fed a forage-dominated diet with minimal concentrate allowance. We hypothesized changes in microbiota structure as well as altered SCFA profiles in various niches of the rumen in response to ZEN. Besides bacteria, protozoa play as well a key role in ruminal mycotoxin biodegradation, in particular regarding ZEN [11]. By benefiting from this biodegradation ability, the abundance of these eukaryotes was further expected to increase after the ZEN dosing.

## 2. Results

### 2.1. Feed Intake and Zearalenone Exposure Level

The total DM intake was constant during the three experimental days (*p* = 0.82), being 11.5, 10.8, and 11.3 kg/cow/day for baseline, ZEN-challenge day, and post-challenge day, respectively (standard error of the mean = 0.88 kg). The ZEN concentration of the day of ZEN-challenge was 10.7 mg/kg for the ZEN-contaminated concentrate, whereas the forages did not contain ZEN, and the uncontaminated concentrate contained 0.104 mg native ZEN/kg (Supplementary Table S1; Gruber-Dorninger et al. [7]). Cows consumed 1.5 kg uncontaminated concentrate plus 0.5 kg ZEN-contaminated concentrate so that the real average ZEN intake during the ZEN-challenge day was 0.156 + 5.350 = 5.506 mg/cow, meaning that the ZEN concentration in the total diet was 0.510 mg/kg DM or 0.449 mg ZEN/kg feed with 88% DM (or 12% moisture). The ZEN intake during baseline and post-ZEN-challenge day was similarly low (0.208 mg/cow), and the ZEN level in the diet was only 0.018 mg/kg DM (or 0.016 mg/kg feed with 88% DM).

### 2.2. Changes in Concentrations of Zearalenone and Its Metabolites in the Rumen

**Figure 1:**
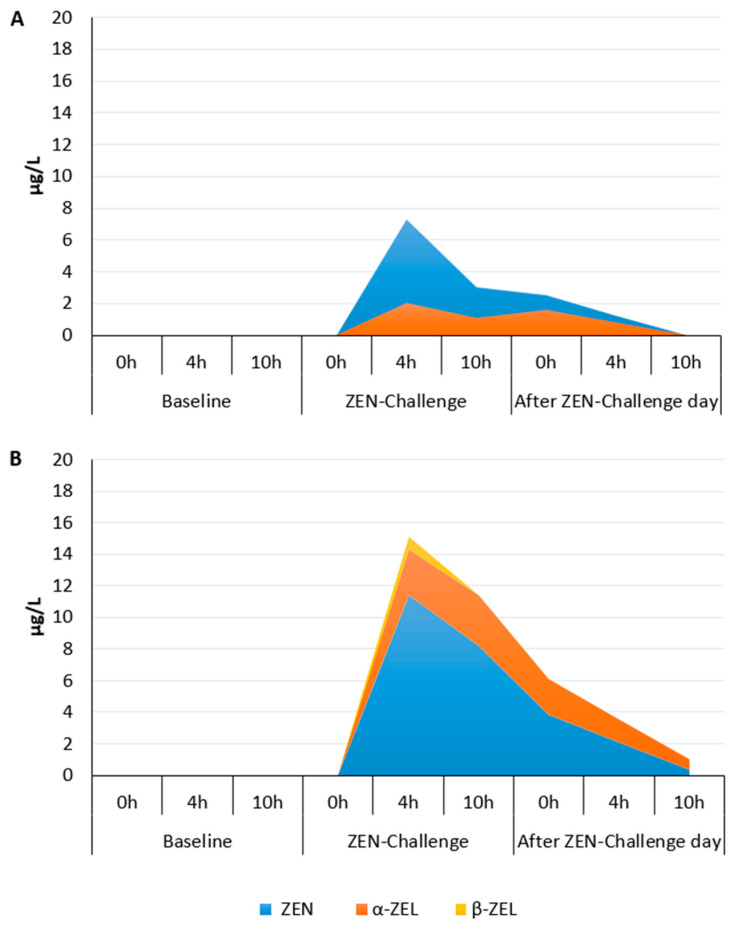
Concentrations of zearalenone (ZEN) and its metabolites (α-ZEL, α-zearalenol; β-ZEL, β-zearalenol) in free rumen liquid (**A**) and particle-associated rumen liquid (**B**) of dairy cows during baseline, oral ZEN-challenge, or the day post-ZEN-challenge, measured at 3 different time points after the morning feeding (adapted from Gruber-Dorninger et al. [7]).

The concentrations of ZEN and its metabolites at the respective time points after morning feeding were adapted from the companion paper Gruber-Dorninger et al. [7], where the complete dataset is fully presented. During the baseline period, no ZEN, α-ZEL, or β-ZEL were found in the rumen (Figure 1). On the ZEN-challenge day, high concentrations of ZEN and its metabolite α-ZEL were measured in PARL (11.4 and 2.8 µg/l, respectively) and FRL (7.2 and 2.1 µg/L, respectively) at 4 h post-feeding, but their concentration decreased steadily thereafter, yet being even measured at 10 h of the post-challenge day in the PARL, i.e., 34 h after ZEN dosage. Concentrations of the ZEN biodegradation product β-ZEL were only measured in the PARL after the ZEN exposure.

### 2.3. Prokaryotic Microbiota Composition in the Rumen

The sequencing dataset comprised, in total, 2,850,469 reads after quality filtering and removal of contaminants that were assigned to 11,447 ASV, where 92.4% could be assigned up to the genus level. The phyla Firmicutes, Bacteroidetes, and Patescibacteria (candidate superphylum) were most abundant accounting for 90.2% of the microbial communities, and *Christensenellaceae* R-7 group, *Ruminococcaceae* NK4A214 group, *Prevotella* 1, *Rikenellaceae* RC9 gut group, and *Ruminococcus* 1 represented the top five genera (Supplementary Table S2).

Regarding alpha diversity in the FRL (Table 1), the number of observed ASV was influenced by the interaction of ZEN challenge and hour (*p* = 0.01). Shannon (*p* = 0.03) and InvSimpson (*p* < 0.01) indices were both decreased with ZEN challenge. Additionally, both indices showed lower values at 10 h than 4 h with 0 h not differing from other time points (*p* = 0.03 and *p* = 0.01 for Shannon and InvSimpson, respectively). In PARL (Table 1), an interaction was observed for Shannon index with 0 h being higher than 4 h and 10 h on baseline day, but 0 h and 4 h being higher than 10 h on ZEN-challenge and post-challenge days (*p* = 0.03). 

**Figure 2:**
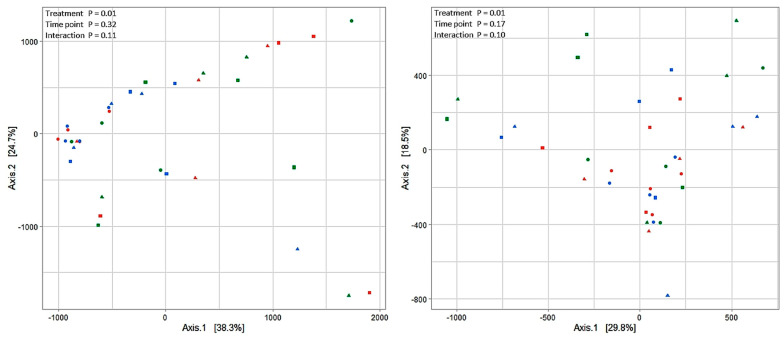
Changes in prokaryotic community composition in free rumen liquid (**left**) or in particle-associated rumen liquid (**right**) visualized as a principal co-ordinate analysis using Aitchison metrics. Different shapes illustrate treatments (● baseline; ▲ ZEN-challenge day; and ■ the after-day ZEN-challenge) and different colors indicate sampling hours (0 h = red; 4 h = blue; and 10 h = green). The percentage of variation explained is indicated on the respective axes.

The analysis of the β-diversity structure showed a separation of samples of ZEN-challenge and post-challenge days from baseline samples along axis 1 for FRL (*p* = 0.01) and PARL (*p* = 0.01; Figure 2). In contrast, sampling time point had no influence on the β-diversity structure in FRL (*p* = 0.32) and PARL (*p* = 0.17), which also applied for the interaction of the two factors (*p* = 0.11 and *p* = 0.10 in FRL and PARL, respectively).

The differential abundance analysis of the FRL revealed the *Bacteroides pectinophilus* group was lower on baseline than on ZEN-challenge day (coefficient = 3.06) and ZEN challenge and post-challenge day (coefficient = 5.21). The Z20 cluster was higher on baseline day than on post-challenge day (coefficient = −2.26), whereas *Succinivibrionaceae* UCG-002 was lower on baseline than on ZEN-challenge day (coefficient 2.23). Regarding sampling time points, *Ruminobacter* was lower at 0 h and 4 h than at 10 h (each coefficient = 2.08). The genera *Pediococcus*, *Weissella*, *Sphingomonas*, *Methylobacterium*, and the *Allorhizobium*-*Neorhizobium*-*Pararhizobium*-*Rhizobium* clade were lower at 0 h than at 4 h (coefficients ≥ 2.14) and 10 h (coefficients ≥ 2.08), while also 4 h were lower than at 10 h (coefficients ≥ 2.17). In the PARL fraction, the *Bacteroides pectinophilus* group was lower on baseline day than on ZEN-challenge day (coefficient = 3.32) and post-challenge day (coefficient = 5.06). Additionally, the *Bacteroides pectinophilus* group, *Ruminobacter* and the *Allorhizobium*-*Neorhizobium*-*Pararhizobium*-*Rhizobium* clade was more abundant at 10 h than at 0 h and 4 h (each coefficient = 2.40, 2.34 and 2.83, respectively).

### 2.4. Absolute Abundances of Microbial Groups in the Rumen

The absolute abundances of total bacteria, archaea, protozoa, and anaerobic fungi for both fractions are presented in Table 2. In the FRL, the sampling time point showed an effect on protozoa with increased abundances from 0 h to 4 h and 10 h after morning feeding (*p* < 0.01), whereas anaerobic fungi increased from 0 h to 4 h but were at the same level as before morning feeding after 10 h (*p* < 0.01). Additionally, we observed a treatment effect on anaerobic fungi that decreased from baseline to ZEN-challenge and post-challenge days (*p* < 0.01). Regarding PARL, an interaction (*p* = 0.04) was observed for archaea with higher abundances at 0 h than at 4 h and 10 h on baseline but higher abundances at 0 h than at 10 h and 4 h. Archaea tended to decrease after morning feeding (*p* = 0.08). The protozoa in PARL were affected by ZEN challenge with a higher abundance on the post-challenge day than at baseline with ZEN-challenge day being intermediate (*p* = 0.02). The bacterial abundance was neither affected nor tendentially different in both fractions (each *p* > 0.10).

### 2.5. Short-Chain Fatty Acid Profiles in the Rumen

The total SCFA concentrations and individual SCFA proportions for FRL and PARL are presented in Table 3. Several interactions of treatment and time point were present in both fractions. In the FRL, iso-butyrate decreased after 4 h from baseline, but increased from 0 h to 4 h and then decreased from 4 h to 10 h to the level of 0 h on ZEN-challenge and post-challenge day. Additionally, iso-butyrate proportions at time points 0 h and 4 h were higher at baseline than on post-challenge day (*p* = 0.02). The iso-valerate at 0 h was higher for baseline than for ZEN-challenge and post-challenge days (*p* = 0.04). For caproate, 0 h proportions were lower than 4 h and 10 h from baseline and post-challenge day, whereas on ZEN-challenge day, caproate differed for all time points with lowest proportions at 0 h, followed by 4 h, and then 10 h. In the PARL, total SCFA concentrations were lowest before morning feeding on all days but maintained a low level after 4 h from baseline, whereas they increased after 4 h on ZEN-challenge and post-challenge days. Similarly, SCFA concentrations at 4 h were higher on ZEN-challenge and post-challenge days when compared to baseline (*p* = 0.03).

An effect of treatment was observed in both fractions for total SCFA (each *p* = 0.02) as well as proportions of iso-butyrate (*p* = 0.03 and *p* = 0.01 for FRL and PARL, respectively) and caproate (*p* < 0.01 and *p* = 0.01 for FRL and PARL, respectively). Thereby, total SCFA and caproate decreased from baseline to ZEN-challenge and post-challenge days, whereas iso-butyrate showed the opposite trend. Additionally, the acetate proportion tended to be higher at baseline compared to ZEN-challenge and post-challenge days in both fractions (each *p* = 0.06), whereas propionate and n-valerate tended to show the opposite direction (each *p* = 0.06 and each *p* = 0.08 for FRL and PARL, respectively). The n-butyrate proportion tended to be lower at baseline than on ZEN-challenge and post-challenge days for FRL only (*p* = 0.08).

For both fractions, sampling time point affected the total SCFA (each *p* = 0.01) and individual SCFA (each *p* < 0.01) with lower levels of total SCFA as well as proportions of propionate, n-butyrate, and n-valerate before morning feeding than 10 h after, whereas proportions of acetate, iso-butyrate, iso-valerate, and caproate were higher before morning feeding than 10 h after.

### 2.6. Correlation Analysis

**Figure 3:**
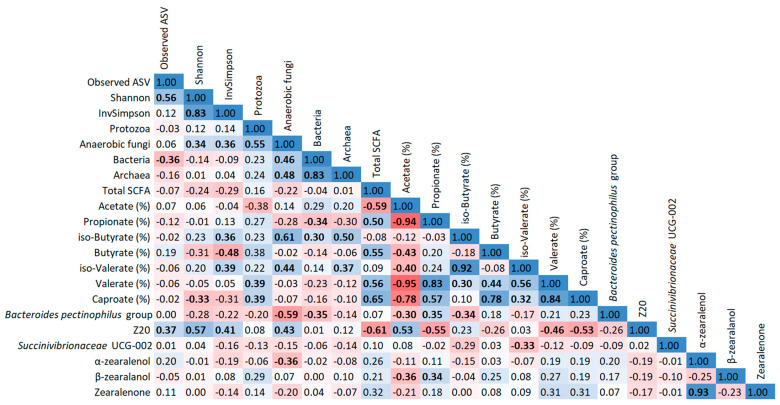
Heatmap illustrating Spearman correlation coefficients between differently abundant genera, qPCR data, alpha diversity indices, SCFA profiles, as well as zearalenone and its metabolites in free rumen liquid. Statistically significant correlations (*p* ≤ 0.05) are marked in bold print. Red coloration indicates a negative correlation; blue coloration indicates a positive correlation. The coloration intensity refers to the correlation coefficient.

**Figure 4:**
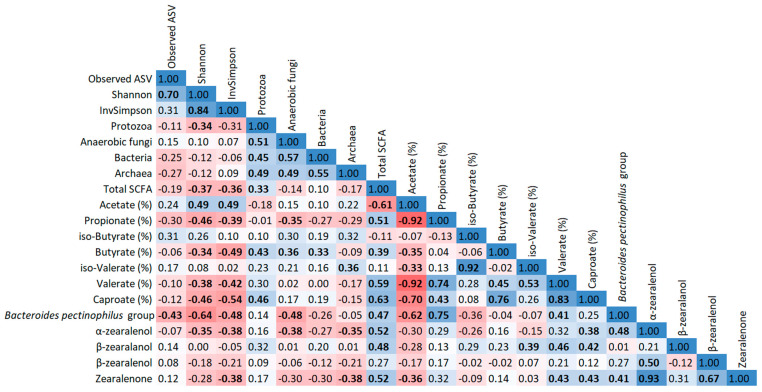
Heatmap illustrating Spearman correlation coefficients between differently abundant genera, qPCR data, alpha diversity indices, SCFA profiles, as well as zearalenone and its metabolites in particle-associated rumen liquid. Statistically significant correlations (*p* ≤ 0.05) are marked in bold print. Red coloration indicates a negative correlation; blue coloration indicates a positive correlation. The coloration intensity refers to the correlation coefficient.

The correlation analysis revealed many statistically significant correlations (*p* ≤ 0.05) between differently abundant genera, qPCR data, alpha diversity indices, SCFA profiles, as well as zearalenone and its metabolites that were visualized in heatmaps for both fractions (Figure 3 and Figure 4). Several correlations can be classified as strong, i.e., r ≥ 0.70 or r ≤ 0.70 [12]: in PARL, the *Bacteroides pectinophilus* group was positively correlated with propionate proportion. Besides, total bacteria and archaea showed a strong positive correlation in FRL, which was also found for Shannon and InvSimpson indices in both fractions. Additionally, Shannon was also positively correlated with observed ASV in PARL. Regarding SCFA, acetate was negatively correlated with propionate, n-valerate, and caproate in both fractions, whereas propionate also showed a positive correlation with n-valerate in both fractions. Similarly, caproate was positively correlated with n-butyrate and n-valerate, and also iso-acids were positively correlated in both FRL and PARL. Additionally, ZEN was positively correlated with α-ZEL in both fractions.

## 3. Discussion

The present study analyzed the short-term impact of a single oral ZEN bolus on the microbiota and fermentation pattern in the rumen of dry Holstein cows fed a forage-rich diet with 2 kg concentrate allowance, as it is commonly the case in pasture-dominated and low input dairy production systems. The level of ZEN fed during the challenge day (0.449 mg ZEN/kg feed with 88% DM) was slightly below the current EU guidance value for ZEN in feedstuffs for dairy cows, which is 0.5 mg/kg relative to feed with a moisture content of 12% [13].

We hypothesized an altered SCFA profile as well as a distinct microbiota structure with increasing abundance of protozoa in response to ZEN exposure. It is noteworthy that no differences in feed intake were found during the experiment and the observed effects should, therefore, be associated with the mycotoxin intervention. The present findings on the prokaryotic communities showed a diminished alpha diversity after ZEN exposure in FRL that maintained also the following day, while Shannon and InvSimpson indices were negatively correlated with ZEN and α-ZEL in PARL. Similarly, the baseline was separately clustering from the day of ZEN exposure and the day after in the PCoA plots of both fractions, therefore, confirming the set-up hypothesis. At the genus level, *Succinivibrionaceae* UCG-002 increased on the day of ZEN exposure in FRL, which may be related to the slightly higher propionate proportion since this genus harbors succinate producers, i.e., an important metabolic intermediate of ruminal propionate formation [14]. However, no significant correlation was found for those two variables. Overall, the alterations observed in the ruminal SCFA profile may be classified as of minor degree. The ZEN treatment induced a marginally closer ratio of acetate to propionate along with decreasing iso-butyrate but increasing caproate proportions in both fractions, whereas increased acetate and iso-butyrate proportions were observed after ZEN exposure of cows fed a moderate-concentrate diet [4]. Thus, concentrate allowance in the diet may determine the direction of the ZEN impact on the ruminal fermentation pattern.

Interestingly, the *Bacteroides pectinophilus* group increased after ZEN exposure in both fractions, a taxon that is hardly described in rumen research. When very cautiously extrapolating intestinal microbiota data from rodents, the *Bacteroides pectinophilus* group could be an indicator of a disturbed microbial community in the gut [15], but the need for further clarification on the role of this microbial group during mycotoxin exposure in the rumen is explicitly emphasized. In contrast, the Z20 cluster belonging to *Oligosphaeraceae* declined in response to ZEN, a bacterial group that is also marginally recognized in rumen research but seems to react sensitively against this mycotoxin. The Z20 cluster as well as the other aforementioned genera were not affected during a comparable ZEN challenge with cows fed a 40% concentrate diet [4], thus, as postulated in regards to the SCFA profile, the concentrate inclusion level seems to be an interacting factor modulating the impact of ZEN on the bacterial community in the rumen. In general, the differential abundance analysis revealed more changes of the bacterial community in FRL than in PARL, suggesting the bacteria of the solid fraction are less responsive to ZEN, particularly when considering that ZEN and its metabolites persist for a longer time in the ruminal fiber mat than in FRL [7]. Indeed, higher levels of ZEN and its metabolites α-ZEL and β-ZEL were present in the PARL than in FRL, which is likely caused by a higher microbial activity and slower washout, and the concentrations remained high in PARL also during the post-challenge day. Hereby, the varying concentrations of ZEN, α-ZEL, and β-ZEL beg the question whether the rumen microbes react sensitively towards this original mycotoxin or its metabolites. In regards to the host animal, ruminal ZEN biodegradation is actually not a detoxification process *per se* but can result in the emergence of more estrogenic metabolites, i.e., α-ZEL that is 60 times as potent as ZEN, whereas β-ZEL possesses only 20% of the estrogenic potency of ZEN [16]. Transferring this scheme to the microbial community in the rumen, it is plausible that ZEN and its metabolites have varying impacts on the members of the rumen microbiota, which needs to be investigated for ZEN and each metabolite in future research.

The differences in bacterial abundances observed between sampling time points, however, may rather derive from the cows’ diurnal eating pattern than from ZEN exposure, such as the increases of *Weissella* or the *Allorhizobium*-*Neorhizobium*-*Pararhizobium*-*Rhizobium* clade from 0 h to 10 h may likely originate from the fed silage e [17], and hence strongly changed during the day course. Likewise, the changes in anaerobic fungi abundances observed between sampling time points followed a typical pattern in daily fed ruminants [18].

The absolute abundance of bacteria was not altered by the ZEN exposure and remained stable during the experiment in both the FRL and PARL. Therefore, the increase of total SCFA concentrations observed in both fractions on the day of ZEN exposure and the day after could not be explained by a quantitative increase of bacteria but should arise from differences in the relative bacterial composition and their activity coupled with changes of the other microbial groups. Indeed, apart from the bacterial community, the present study revealed an increased abundance of protozoa in PARL one day after the mycotoxin challenge when compared to control day, as well as a trend for more protozoa already on the day of mycotoxin exposure than on the control day. Protozoa possess a key role in ruminal mycotoxin degradation, which is particularly true for ZEN [11], and the present qPCR data support the hypothesis that protozoa of the PARL benefit from their biodegradation ability and consequently increase after ZEN exposure in the rumen—potentially also due to tolerating higher levels of β-ZEL. In this context, the longer presence of ZEN and its associated metabolites in the ruminal fiber mat [7] may explain why the promoting effect of ZEN on protozoa was detected in PARL only. Furthermore, it may be noted that the higher protozoa concentration after ZEN exposure suggests a specific sensitivity against *Fusarium* toxins as protozoal growth was substantially impaired by T-2 toxin in vitro [19] but obviously not by ZEN.

The rumen archaea appeared to be tolerant to ZEN and no effects were observed in FRL. Only in PARL, archaea decreased 4 h after ZEN dosage when compared to their abundance on the control day, which was also apparent from the negative correlation of archaea and ZEN in this fraction. This decline, however, was not lasting and was already diminished 10 h after the ZEN exposure—in fact, PARL-associated archaea rather showed an overcompensation as their abundance was higher at 10 h on the day after ZEN exposure than on the control day, which may be related to their intertwining with ruminal protozoa but needs further investigation at a genus level [20].

Regarding anaerobic fungi, the mycotoxin exposure resulted in a decrease in FRL, the fraction that primarily comprises flagellated fungal zoospores that are released from rhizoidal rumen fungi of the solid fraction [18,21]. Moreover, fairly negative correlations of anaerobic fungi and α-ZEL were present in both FRL and PARL, indicating that rather this ZEN-metabolite than the original mycotoxin affected this microbial group in the rumen. It is, therefore, conceivable that α-ZEL impaired the fungal reproduction meaning fewer zoospores were released by anaerobic fungi colonizing particles in the fiber mat and would consequently imply that, despite the present observation on reduced fungal abundance was made in FRL, the negative impact of α-ZEL on rumen anaerobic fungi would actually have occurred in the solid fraction. Similarly, the fairly negative correlation of anaerobic fungi with propionate proportion in PARL, which is in line with early results made in sheep [22], would also fit the concept of α-ZEL negatively affecting the anaerobic fungi of the solid rumen fraction. It is noteworthy, however, that this assumption is difficult to prove since it is methodologically not possible to discriminate from which stage of the fungal life cycle the DNA of FRL and PARL originates.

## 4. Conclusions

The single oral ZEN bolus had short-term implications on the microbial community and fermentation pattern in the rumen of dry Holstein cows fed a forage-rich diet with minimal concentrate allowance. The rumen bacteria were altered at a compositional level but not quantitatively, whereas protozoa may have taken advantage of their high detoxification capacity and, therefore, increased in the PARL fraction. Hereby, it mainly remains to be solved whether ZEN itself or its metabolites or their synergistic effect were responsible for the alterations observed. Archaea seemed to be less affected by ZEN, whereas anaerobic fungi could be compromised by α-ZEL emerging after ZEN biodegradation. The changes in SCFA profiles were of minor extent and indicated no detrimental effect on rumen fermentation. Consequently, a characterization of the ruminal eukaryotic communities in future studies may help to better understand the impact of ZEN and its metabolites in the rumen and substantiate the present findings. Therefore, it should be further explored to what extent ZEN also affects animal health when ruminants are fed forage-dominated diets.

## 5. Materials and Methods

The present study was approved by the Institutional Ethics and Animal Welfare Committee of the University of Veterinary Medicine Vienna and the national authority according to §26 of the Law for Animal Experiments, Tierversuchsgesetz 2012-TVG (GZ: 68.205/0156-WF/V/3b/2017, 30 August 2017).

### 5.1. Animals and Feeding

The experiment was conducted at the VetFarm of the University of Veterinary Medicine Vienna in Pottenstein, Austria. Four dry Holstein cows fitted with rumen cannulas were housed in a free-stall barn equipped with 12 deep litter cubicles (2.6 m × 1.25 m) with ad libitum access to water and salt lick stones. The present paper is part of a larger research study, and the results dealing with kinetics of ZEN degradation in the bovine gastrointestinal tract and the role of a ZEN-degrading enzyme were published earlier [7], in which experimental setup, animals, and feeding methods are reported in detail. In brief, the cows were fed a forage-dominated basal diet *ad libitum*, consisting of grass silage and hay (50:50 on dry matter basis), plus 2 kg of concentrate per day (Supplementary Table S1). The forage diet was prepared daily, and fresh feed was offered to the cows in the morning (08:30 a.m.) and afternoon (03:30 p.m.), whereby the concentrate was distributed during the day in 4 portions of 500 g each (08:30 a.m., 11:30 a.m., 02:30 p.m., and 05:30 p.m.). Feed intakes were recorded for each cow via individual feeding troughs equipped with electronic weighing scales and computer-regulated access gates (Insentec B.V., the Netherlands). The cows were adapted to the basal diet and the feeding troughs for several days before the experiment.

### 5.2. Experimental Design and Oral ZEN Challenge

The experiment was conducted as a longitudinal feeding trial with cows being experimental units, and each cow served as its own control with day 1 being performed as baseline with multiple measurements. On the baseline day, all cows were fed the 500 g uncontaminated concentrate shortly before they received their morning feeding. On the second day, the cows were challenged by a one-time bolus of 5 mg ZEN spiked on 500 g concentrate (10 mg ZEN/kg), which was fed to the cows shortly before the morning diet. The ZEN was obtained from a *Fusarium graminearum* culture, and the procedure has been described in detail by Gruber-Dorninger et al. [7]. The day after the ZEN-challenge, the cows again received 500 g of uncontaminated concentrate before the morning diet to evaluate the effects of single dose ZEN exposure over 34 h post-challenge.

### 5.3. Ruminal Samplings

During baseline, ZEN exposure, and post-exposure days cows were sampled for free rumen liquid (FRL), which was collected via the rumen cannula before morning feeding, i.e., 0 h, 4 h, and 10 h after the morning feeding using single-use 20 mL syringes. To obtain particle-associated rumen liquid (PARL), solid rumen digesta samples were taken from the rumen mat of the dorsal sac at the same hours as FRL and squeezed through three layers of medical gauze (Wilhelm Weisweiler GmbH & Co. KG, Münster, Germany). Aliquots for DNA extraction were placed in cryotubes and snap-frozen in liquid nitrogen before being stored at −80 °C until further processing. The aliquots for SCFA determination were stored in 2 mL Eppendorf tubes at −20 °C until analysis. Feed samples, i.e., grass silage, hay, and concentrate, were collected before the baseline day and after post-ZEN-challenge day and also stored at −20 °C until the analysis.

### 5.4. Analysis of Feedstuffs

The feed samples were dried at 65 °C in a forced-air oven for 48 h and subsequently ground through a 1 mm screen (Ultra Centrifugal Mill ZM 200, Retsch, Haan, Germany). Subsequently, the nutrient analyses were conducted in accordance with the Association of German Agricultural Analytic and Research Institutes [23]. The DM concentration was determined by oven-drying at 103 °C for at least 4 h (method 3.1). The ash concentration was analyzed by combustion in a muffle furnace overnight at 580 °C (method 8.1). The crude protein was determined using the Kjeldahl method (method 4.1.1) and ether extract via the soxhlet extraction system (method 5.1.2). Determination of neutral and acid detergent fiber was conducted in accordance with methods 6.5.1 and 6.5.2, respectively, using the Fiber Therm FT 12 (Gerhardt GmbH & Co. KG, Germany). Therefore, neutral detergent fiber was assayed with a heat stable α-amylase and both fiber fractions were expressed exclusive of residual ash. Additionally, feeds were also analyzed for ZEN using HPLC-MS/MS with the methods and conditions described in Gruber-Dorninger et al. [7].

### 5.5. DNA Extraction and Prokaryotic 16S rRNA Gene Sequencing

The DNA extraction was performed from FRL and PARL samples from the rumen using the DNeasy PowerSoil Kit (Qiagen, Hilden, Germany) in a Qiacube instrument (Qiagen, Hilden, Germany), as described in Klevenhusen et al. [24], with an additional bead-beating step for PARL samples. The 16S rRNA gene sequencing was performed on the Illumina MiSeq platform (Microsynth AG, Balgach, Switzerland). Targeted amplification of the hypervariable region V3–V4 of bacterial 16S rRNA gene (2 × 250 bp) was performed using the primers 341F_ill (5-CCTACGGGNGGCWGCAG-3) and 802R_ill (5-GACTACHVGGGTATCTAATCC-3) of Herlemann et al. [25]. Multiplexed libraries were constructed by ligating sequencing adapters and indices onto purified PCR products using the Nextera XT Sample Preparation Kit (Illumina, Balgach, Switzerland). Primers were trimmed and corresponding overlapping paired-end reads were stitched by Microsynth (Microsynth AG, Balgach, Switzerland).

### 5.6. qPCR Analysis

Absolute quantification of total bacteria, archaea, protozoa, and anaerobic fungi was performed by qPCR analysis on a Mx3000P thermocycler (Agilent Technologies, Santa Clara, CA, USA), including melting curve analysis to ensure primer specificity, as described in detail in Klevenhusen et al. [24]. Briefly, all amplification reactions were run in duplicate with 1 µL of genomic DNA and a final volume of 25 µL in EvaGreen^®^-based qPCR assays. The standard curves were constructed using the primer sets and reaction conditions given in Table 4. The respective gene copy numbers were determined by relating the quantification cycle values to standard curves and final copy numbers per milliliter of ruminal fluid were calculated using the equation of Li et al. [26].

### 5.7. Analysis of Short-Chain Fatty Acids, Zearalenone and Its Metabolites

Sample preparation and analysis of concentrations of acetate, propionate, n-butyrate, iso-butyrate, n-valerate, iso-valerate, and caproate were performed as described before in Poier et al. [31]. In brief, samples of FRL and PARL were centrifuged at 20,000× *g* for 25 min at 4 °C after thawing at room temperature. Then, 0.6 mL of supernatant was transferred into a fresh tube and 0.2 mL of HCl (1.8 mol/L) was added, followed by 0.2 mL of 4-methylvaleric acid (Sigma-Aldrich Co. LLC., St. Louis, MO, USA) that was used as internal standard. The mixture was centrifuged again for 25 min at 20,000× *g* and 4 °C, and the clear supernatant was used for SCFA determination on a gas chromatography device (GC Model 8060 MS 172 DPFC, No.: 950713, Fisons, Italy).

The analysis of ZEN and its metabolites α-ZEL and β-zearalenol (β-ZEL) in feed and rumen samples was performed using a HPLC system (Agilent Technologies, Waldbronn, Germany) coupled to a 5500 QTrap mass spectrometer equipped with an electrospray ionization source (SCIEX, Foster City, CA, USA), as described in detail by Gruber-Dorninger et al. [7].

### 5.8. Bioinformatic and Statistical Analysis

The sequencing data was processed with the software package Quantitative Insights into Microbial Ecology (QIIME2 v2020.2; [32]). Therefore, read quality was inspected using FASTQC for demultiplexed Illumina fastq data with the PHRED score offset of 33 and sequences were merged with VSEARCH [33] before being quality filtered using the q-score-joined plugin and 20 as a minimum acceptable PHRED score. Denoising into amplicon sequence variants (ASV) was performed using Deblur [34] and representative sequences and feature tables were filtered to remove mitochondria or chloroplast sequences. The resulting filtered ASV were aligned with mafft [35] and a phylogeny was constructed with FastTree2 [36]. Taxonomy was assigned to ASV using a classify-sklearn naïve Bayes taxonomy classifier trained with the 341F/802R primer set against the SILVA 132 99% OTUs reference sequences [37]. Subsequently, the filtered feature table, rooted tree, and taxonomy were imported in Rstudio v14.1717.

The statistical analysis was conducted separately for FRL and PARL. Thereby, data sets of SCFA (total SCFA and proportions of individual SCFA) and alpha diversity indices as well as feed intake were analyzed in SAS v9.4 (SAS Institute Inc., USA) using proc univariate to validate normal distribution by the Shapiro–Wilk’s normality method. Subsequently, an ANOVA was performed using proc mixed with the following model:

Y_ijk_ = µ + d_i_ + t_j_ + (d × t)_ij_ + c_k_ + e_ijk_

where µ is the mean, d_j_ is the fixed effect of the ZEN challenge, t_j_ is the fixed effect of sampling hour (not applicable for feed intake), c_k_ is the random effect of individual cow, and e_ijk_ is the residual error. Measurements obtained for the same cow but at different hours were considered as repeated measurements within treatment and cow, and differences between least-squares means were tested via the pdiff option. All results are presented as least-square means with the largest standard error of the mean. The significance level was defined at *p* ≤ 0.05 and trends were declared at 0.05 < *p* < 0.10 for all statistical analyses.

The multivariate data analysis was done in Rstudio using the packages qiime2R v0.99.6, phyloseq v1.24.2, vegan v2.5-7, MaAsLin2 v1.5.1, and ggplot2 v3.3.3 [38,39,40,41,42]. The alpha diversity indices observed ASV, Shannon, and InvSimpson were calculated in Rstudio and then transferred to SAS v9.4 for statistical analysis using the model mentioned above. For beta diversity analysis, Aitchison metrics were used to perform principal co-ordinate analysis (PCoA) and sample groupings in the PCoA were tested for significance by the *adonis* function [43]. Regarding the differential abundances, changes at genus level were considered as relevant if coefficient was <−2.00 or >2.00 and Benjamini–Hochberg false discovery rate-adjusted q-values ≤ 0.05. Additionally, Spearman correlation coefficients between data sets of SCFA, qPCR analysis, alpha diversity indices, and differentially abundant genera were calculated using proc corr in SAS v9.4 (SAS Institute Inc., Cary, NC, USA) and subsequently visualized in heatmaps. In order to uniformly report the strengths of the coefficients, they were ranked according to Akoglu [12].

## Figures and Tables

**Table 1:** Alpha diversity indices in free rumen liquid (FRL) and particle-associated rumen liquid (PARL) measured at different hours after feeding.

Treatment	Baseline				ZEN-Challenge Day				After ZEN-Challenge Day					*p*-Values ^2^		
Time after Feeding	0 h	4 h	10 h	Mean	0 h	4 h	10 h	Mean	0 h	4 h	10 h	Mean	SEM ^1^	Trt	Time	Trt × Time
FRL																
Observed ASV	3918 ^A ab^	3983 ^a^	3556 ^b^	3819	3136 ^Bb^	3760 ^a^	3720 ^a^	3539	4182 ^Aa^	3683 ^b^	3813 ^ab^	3892	187	0.23	0.61	0.01
Shannon	7.32	7.34	7.18	7.28 ^X^	7.03	7.21	7.08	7.11 ^Y^	7.13	7.16	7.03	7.11 ^Y^	0.07	0.03	0.03	0.54
InvSimpson	464	456	352	424 ^X^	241	352	207	267 ^Y^	226	367	234	276 ^Y^	49.1	<0.01	0.01	0.60
PARL																
Observed ASV	3236	3165	3190	3197	3337	3509	3089	3312	3067	3189	2958	3071	114	0.15	0.03	0.26
Shannon	7.13 ^a^	7.08 ^b^	7.08 ^b^	7.10	7.11 ^a^	7.07 ^a^	6.97 ^b^	7.05	7.09 ^a^	7.06 ^a^	6.92 ^b^	7.02	0.04	0.30	<0.01	0.03
InvSimpson	383	359	343	362	352	314	254	307	385	343	247	325	22.9	0.10	<0.01	0.09

^1^ Largest standard error of the mean. ^2^ Effect of treatment (Trt), hour (time), and their interaction. In each row, superscript in capitalized letters “A, B” indicate differences (*p* ≤ 0.05) between hours of different treatments and superscript with lowercase letters “a, b” indicate differences (*p* ≤ 0.05) between hours within each treatment, whereas superscript with capitalized letters “X, Y” indicate difference (*p* ≤ 0.05) among means of treatments.

**Table 2:** Absolute abundances (log_10_ gene copies/)mL of total bacteria, archaea, protozoa, and anaerobic fungi in free rumen liquid (FRL) and particle-associated rumen liquid (PARL).

Treatment	Baseline				ZEN-Challenge-Day				After ZEN-Challenge Day					*p*-Values ^2^		
Time after Feeding	0 h	4 h	10 h	Mean	0 h	4 h	10 h	Mean	0 h	4 h	10 h	Mean	SEM ^1^	Trt	Time	Trt × Time
FRL																
Bacteria	6.09	6.11	6.18	6.12	6.18	6.08	6.05	6.10	5.97	6.09	5.94	6.00	0.08	0.19	0.79	0.36
Archaea	4.69	4.82	4.72	4.74	4.75	4.62	4.59	4.65	4.59	4.70	4.52	4.60	0.14	0.39	0.62	0.85
Protozoa	4.14	4.61	4.80	4.51	4.04	4.56	4.63	4.41	3.87	4.36	4.52	4.25	0.14	0.13	<0.01	0.71
Anaerobic fungi	3.76	4.09	3.83	3.89 ^X^	3.51	3.67	3.50	3.56 ^Y^	3.30	3.67	3.46	3.48 ^Y^	0.11	<0.01	<0.01	0.76
PARL																
Bacteria	6.17	6.10	6.16	6.15	6.20	6.12	6.08	6.13	6.16	6.14	6.26	6.18	0.06	0.30	0.33	0.42
Archaea	5.29 ^a^	5.23 ^Aa^	4.85 ^Bb^	5.12	5.18 ^a^	4.90 ^Bab^	4.86 ^Bb^	4.98	5.06	5.06 ^AB^	5.22 ^A^	5.11	0.12	0.27	0.08	0.04
Protozoa	4.43	4.59	4.43	4.48 ^Y^	4.61	4.72	4.52	4.61 ^XY^	4.66	4.72	4.81	4.73 ^X^	0.09	0.02	0.27	0.34
Anaerobic fungi	3.82	4.11	3.90	3.95	4.03	3.94	3.68	3.88	3.77	3.95	4.02	3.91	0.13	0.80	0.28	0.10

^1^ Largest standard error of the mean. ^2^ Effect of treatment (Trt), hour (time), and their interaction. In each row, superscript in capitalized letters “A, B” indicate differences (*p* ≤ 0.05) between hours of different treatments and superscript with lowercase letters “a, b” indicate differences (*p* ≤ 0.05) between hours within each treatment, whereas superscript with capitalized letters “X, Y” indicate differences (*p* ≤ 0.05) among means of treatments.

**Table 3:** Concentrations of total short-chain fatty acids (SCFA; mmol/L) and proportions of individual SCFA profiles (% of total SCFA) in free rumen liquid (FRL) and particle-associated rumen liquid (PARL).

Treatment	Baseline				ZEN-Challenge-Day				After ZEN-Challenge Day					*p*-Values ^2^		
Time after Feeding	0 h	4 h	10 h	Mean	0 h	4 h	10 h	Mean	0 h	4 h	10 h	Mean	SEM^1^	Trt	Time	Trt × Time
FRL																
Total SCFA	67.0	66.4	88.6	74.0 ^Y^	82.7	98.1	100.5	93.8 ^X^	78.7	105.8	98.5	94.3 ^X^	6.88	0.02	0.01	0.30
Acetate	72.3	69.4	69.0	70.2	72.2	68.3	67.7	69.4	71.7	67.8	67.1	68.9	0.74	0.06	<0.01	0.66
Propionate	16.2	17.9	19.1	17.7	16.3	18.8	19.0	18.0	16.8	19.2	19.7	18.5	0.73	0.06	<0.01	0.71
n-Butyrate	8.54	8.89	8.86	8.76	8.82	8.98	9.62	9.14	8.92	9.18	9.60	9.23	0.24	0.08	<0.01	0.07
iso-Butyrate	0.83 ^Aa^	0.91 ^Aa^	0.73 ^b^	0.82 ^X^	0.71 ^Ab^	0.83 ^Aba^	0.75 ^b^	0.76 ^XY^	0.64 ^Bb^	0.78 ^Ba^	0.71 ^b^	0.71 ^Y^	0.04	0.03	<0.01	0.02
n-Valerate	0.80	1.26	1.10	1.05	0.80	1.43	1.43	1.22	0.85	1.42	1.40	1.22	0.08	0.06	<0.01	0.17
iso-Valerate	1.08 ^Ab^	1.28 ^a^	0.89 ^c^	1.08	0.88 ^Bb^	1.19 ^a^	0.98 ^b^	1.02	0.77 ^Bb^	1.15 ^a^	0.99 ^a^	0.97	0.08	0.26	<0.01	0.04
Caproate	0.30 ^b^	0.39 ^Ba^	0.37 ^Ba^	0.35 ^Y^	0.32 ^c^	0.48 ^Ab^	0.57 ^Aa^	0.46 ^X^	0.34 ^b^	0.53 ^Aa^	0.55 ^Aa^	0.47 ^X^	0.03	<0.01	<0.01	0.01
PARL																
Total SCFA	79.7 ^b^	75.3 ^Bb^	94.1 ^a^	83.0 ^Y^	85.8 ^b^	102.9 ^Aa^	97.7 ^a^	95.5 ^X^	91.0 ^b^	108.3 ^Aa^	99.1 ^ab^	99.5 ^X^	6.04	0.02	0.01	0.03
Acetate	72.1	69.2	68.7	70.0	71.9	68.3	67.9	69.4	71.6	67.9	67.1	68.9	0.67	0.06	<0.01	0.69
Propionate	16.3	17.9	19.1	17.8	16.4	18.6	19.3	18.1	16.9	19.0	19.6	18.5	0.73	0.08	<0.01	0.89
n-Butyrate	8.64	9.01	8.92	8.86	8.88	9.12	9.29	9.10	8.85	9.16	9.64	9.21	0.25	0.29	<0.01	0.25
iso-Butyrate	0.82 ^Ab^	0.91 ^Aa^	0.74 ^c^	0.82 ^X^	0.71 ^Bb^	0.83 ^Ba^	0.72 ^b^	0.75 ^Y^	0.65 ^Bb^	0.78 ^Ba^	0.71 ^b^	0.71 ^Y^	0.04	0.01	<0.01	0.05
n-Valerate	0.84	1.30	1.16	1.10	0.85	1.48	1.38	1.24	0.89	1.46	1.43	1.26	0.07	0.08	<0.01	0.47
iso-Valerate	1.08 ^Ab^	1.28 ^a^	0.91 ^c^	1.09	0.88 ^Bb^	1.17 ^a^	0.94 ^b^	1.00	0.79 ^Bc^	1.13 ^a^	0.98 ^b^	0.96	0.07	0.14	<0.01	0.03
Caproate	0.31	0.41	0.41	0.37 ^Y^	0.35	0.53	0.54	0.47 ^X^	0.37	0.57	0.58	0.51 ^X^	0.04	0.01	<0.01	0.27

^1^ Standard error of the mean. ^2^ Effect of treatment (Trt), hour (time), and their interaction. In each row, superscript in capitalized letters “A, B” indicate differences (*p* ≤ 0.05) between hours of different treatments and superscript with lowercase letters “a, b” indicate differences (*p* ≤ 0.05) between hours within each treatment, whereas superscript with capitalized letters “X, Y” indicate differences (*p* ≤ 0.05) among means of treatments.

**Table 4:** Primer pairs and reaction conditions applied for qPCR analysis.

Target	Item	Primer Sequence (5′–3′)	Annealing Temperature (°C)	Primer Concentration (nmol)	Amplicon Size (bp)	Reference
Bacteria	F ^1^	CCTACGGGAGGCAGCAG	61	100	189	[27]
	R ^2^	ATTACCGCGGCTGCTGG				
Archaea	F	CCGGAGATGGAACCTGAGAC	60	100	160	[28]
	R	CGGTCTTGCCCAGCTCTTATTC				
Protozoa	F	GCTTTCGWTGGTAGTGTATT	60	400	233	[29]
	R	CTTGCCCTCYAATCGTWCT				
Anaerobic fungi	F	GAGGAAGTAAAAGTCGTAACAAGGTTTC	60	200	110–115	[30]
	R	CAAATTCACAAAGGGTAGGATGATT				

^1^ Forward. ^2^ Reverse.

## Data Availability

All sequencing data have been submitted to the sequence read archive of the National Center for Biotechnology Information and can be accessed under the accession number PRJNA911950.

## Supplementary Materials

The following supporting information can be downloaded at: https://www.mdpi.com/article/10.3390/toxins15030185/s1. Table S1: Chemical composition and ZEN levels of diet components fed to Holstein cows (in % of dry matter unless not stated otherwise, ± standard deviation; Gruber-Dorninger et al. [7]); Table S2: Relative abundances at phylum and genus level.
