# Supplementary material for: Zearalenone and Its Emerging Metabolites Promptly Affect the Rumen Microbiota in Holstein Cows Fed a Forage-Rich Diet
Source: Toxins (Basel). 2023 Feb 28;15(3):185. doi: 10.3390/toxins15030185 (PMC10053043; doi:10.3390/toxins15030185)
Supplement: Supplementary file 1 [file toxins-15-00185-s001.zip › Supplementary_Table_S1.pdf]

toxins

MDPI

Supplementary Materials: Zearalenone and Its Emerging Metabolites Promptly Affect the Rumen Microbiota in Holstein Cows Fed a Forage-Rich Diet

Thomas Hartinger, Iris Kröger, Viktoria Neubauer, Johannes Faas, Barbara Doupovec, Dian Schatzmayr and Qendrim Zebeli

Table S1. Chemical composition and ZEN levels of diet components fed to Holstein cows (in % of dry matter unless not stated otherwise, ± standard deviation; Gruber-Dorninger et al., [7]).

Item	Grass silage hay mix (50:50)	Concentrate
Dry matter (g/kg fresh matter)	54.6 ± 1.08	89.6 ± 0.18
Crude protein	13.2 ± 0.37	11.0 ± 0.55
Ether extract	1.70 ± 0.16	2.76 ± 0.09
Ash	7.49 ± 0.41	7.98 ± 0.28
aNDFom[1]	57.5 ± 1.06	13.7 ± 0.34
ADFom[2]	37.4 ± 0.62	4.39 ± 0.24
Zearalenone[3] (µg/kg)	<LOQ	104

[1]Neutral detergent fiber assayed with a heat stable α-amylase and expressed exclusive of residual ash. [2]Acid detergent fiber expressed exclusive of residual ash. [3]The analyzed zearalenone concentration was 10.7 mg/kg for the ZEN-contaminated concentrate; LOQ, level of quantification.
